# Breastfed 13 month-old infant of a mother with COVID-19 pneumonia: a case report

**DOI:** 10.1186/s13006-020-00305-9

**Published:** 2020-08-06

**Authors:** Yuanyuan Yu, Youjiang Li, Yingying Hu, Bin Li, Jian Xu

**Affiliations:** 1 Department of Obstetrics and Gynecology, The Fourth Affiliated Hospital Zhejiang University School of Medicine, N1 Shangcheng Avenue, Yiwu, 322000 Zhejiang Province China; 2 Department of Clinical Laboratory, The Fourth Affiliated Hospital Zhejiang University School of Medicine, N1 Shangcheng Avenue, Yiwu, 322000 Zhejiang Province China; 3 Department of Infectious Diseases, The Fourth Affiliated Hospital Zhejiang University, School of Medicine, N1 Shangcheng Avenue, Yiwu, 322000 Zhejiang Province China

**Keywords:** COVID-19 pneumonia, SARS-CoV-2, Breastfeeding

## Abstract

**Background:**

In China, mothers with confirmed or suspected COVID-19 pneumonia are recommended to stop breastfeeding. However, the evidence to support this guidance is lacking. There have been relatively few cases reported about direct breastfeeding an infant by a mother with SARS-CoV-2 pneumonia. Therefore, it is necessary to assess the safety of breastfeeding and the possible protective effects of breast milk on infants.

**Case presentation:**

This report analyzes the case of a mother who continued breastfeeding her 13 month-old child when both were diagnosed with confirmed COVID-19 pneumonia. We describe the clinical presentation, diagnosis, treatment, and outcome. The presence of SARS-CoV-2 nucleic acid was determined in maternal serum, breast milk, nasopharyngeal (NP) swabs and feces, and in infant serum, NP swabs and feces. IgM and IgG antibodies against SARS-CoV-2 were assessed in maternal serum and breast milk and in infant serum. SARS-CoV-2 nucleic acid was not detected in the breast milk, and antibodies against SARS-CoV-2 were detected in the mother’s serum and milk.

**Conclusions:**

The present case further confirms that the possibility of mother-to-child transmission about SARS-CoV-2 via breast milk alone was very small, and breast milk is safe for direct feeding of infants.

## Background

In early December 2019, a new coronavirus named SARS-CoV-2 broke out in Wuhan, China, affecting the susceptible population and causing a highly infectious COVID-19 pneumonia [1]. With cases now confirmed in multiple countries and an approximate mortality of 4.0% in China [2], COVID-19 was declared by the World Health Organization (WHO) as a global public health emergency [3]. SARS-CoV-2 spreads primarily through droplets and close contact. According to the recommendations of experts and decision of authorities in China [4, 5], patients with confirmed or suspected COVID-19 pneumonia should stop breastfeeding until recovery. However, it is uncertain whether SARS-CoV-2 can be transmitted through breastmilk. A recent report of nine cases of pregnant women has shown that there is no evidence of vertical transmission [3]. Here, we document for the first time a case of breastfeeding an infant by a SARS-CoV-2 infected mother and describe the clinical presentation, diagnosis, treatment, and outcome. To assess the safety of breastfeeding and the possible protective effects of breast milk on infants, the presence of SARS-CoV-2 nucleic acid was determined in maternal serum, breast milk, nasopharyngeal (NP) swabs and feces, and in the infant’s serum, NP swabs and feces. IgM and IgG antibodies against SARS-CoV-2 were assessed in maternal serum and breast milk and in infant serum.

## Case presentation

The patient was a 32-year-old female, mother of a 13-month-old boy who was directly breastfed since birth with complementary food added at 6 months of age. On January 20, 2020, the patient and her son had a family meal with relatives who returned to Yiwu from Wuhan for the Spring Festival. Two weeks later the patient had nasal congestion, and her son had a fever with a peak temperature of 38.4 °C, dry cough, and nasal congestion. Two days after the onset (February 2, 2020), tests for SARS-CoV-2 nucleic acid performed at the Fourth Affiliated Hospital, Zhejiang University School of Medicine, were positive in both the mother and the son, whereas the patient’s husband had a negative result. The results were confirmed by the Yiwu Center for Disease Control and Prevention. The patient suffered from postpartum depression, felt deeply anxious, and insisted on staying with her child. At the same time, the husband asked to accompany his wife and son due to concerns about the patient’s mental health. To respect the wish of the patient and her family, and after consultation with psychiatrists, the family was treated in the same negative-pressure isolation ward room.

**Fig. 1:**
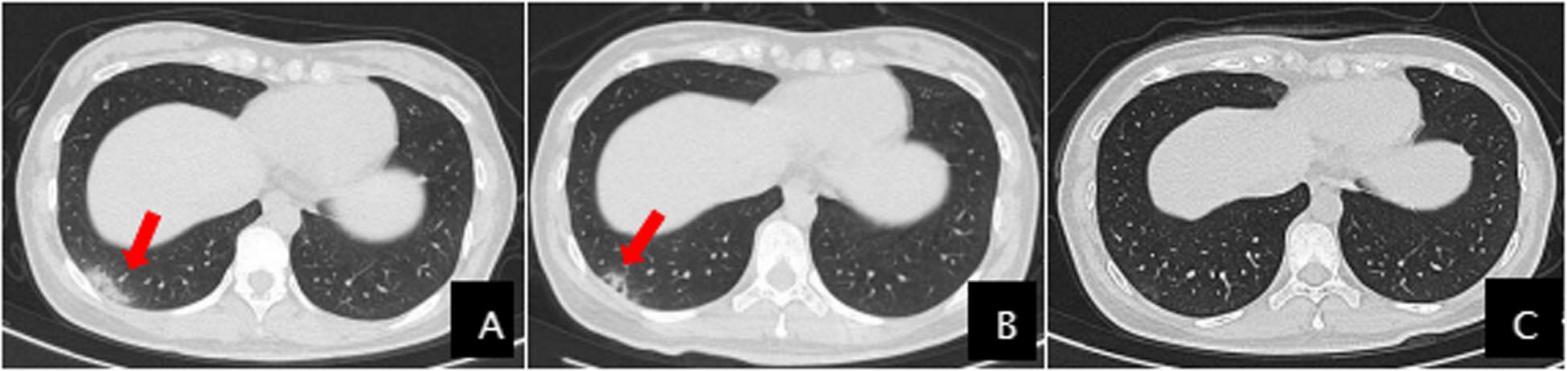
**a-c**: Chest CT plain scans of the mother. **a**. Hospital day 4: density-increased patchy consolidation and ground-glass shadow in the lower lobe of the right lung; **b**. Hospital day 10: strip of high-density shadows in the right lower lobe of the lung with partial absorption; **c**. Hospital day 23: shadows in the right lower lobe of the lung were largely absorbed

On admission, the mother had nasal congestion, but no rhinorrhea, cough, sputum, fever, or fatigue. The physical examination revealed a body temperature of 36.4 °C, respiratory rate of 18 breaths per minute, a pulse of 90 beats per minute, blood pressure of 102/74 mmHg, and oxygen saturation of 98% while breathing ambient air. Lung auscultation indicated no abnormalities. The results of a routine blood tests (complete blood count, liver and renal function), C-reactive protein and chest Xray were normal. Respiratory virus antigen quadruplet test (Influenza A and B virus, respiratory syncytial virus, adenovirus) and the nucleic acid test for Influenza A and B virus were negative. The patient received atomized inhalation of recombinant human interferon α-2b 5 million International Unit (IU) in 2 mL sterilized water twice a day as the antiviral treatment from hospital day one to day 28 and traditional Chinese medicine as a supplemental therapy from hospital day four to day 28. She continued directly breastfeeding four to five times every day. The symptom of nasal congestion improved on hospital day 2, and white blood cell count declined to 2.7 × 10^9^/L, while the lymphocyte count was 0.9 × 10^9^/L. The nasopharyngeal swab specimens were positive for SARS-CoV-2 nucleic acid, but the serum was negative. On hospital day 3, the symptoms of nasal congestion disappeared, not to return. The mother’s body temperature and oxygen saturation without oxygen inhalation were monitored and remained within the normal range, and the patient was in stable condition. On hospital day 4, the plain chest CT scan indicated the presence of density-increased patchy consolidation and ground-glass shadow in the lower lobe of the right lung, and viral pneumonia was the likely diagnosis (Fig. 1a). Subsequently, on hospital day 10, white blood cell and lymphocyte counts returned to a normal level, and liver and renal function and myocardial enzyme tests continued to be normal. During the period of hospitalization, the mother’s serum, milk and feces specimens tested negative for SARS-CoV-2 nucleic acid, but the nasopharyngeal swabs were repeatedly positive. Chest CT scans on hospital days 10 and 23 showed the gradual clearing of the lungs. (Fig. 1b, c). On hospital days 9 and 25 after admission, the breastmilk tests yielded a positive result for SARS-CoV-2 IgG and negative for IgM. Similarly, the maternal serum was positive for SARS-CoV-2 IgG and negative for IgM on hospital days 16 and 20. On hospital day 28, the patient was discharged as three consecutive nasopharyngeal swabs were negative for SARS- CoV-2 nucleic acid. The major laboratory results of the patient are listed in Table 1.

**Table 1:** Clinical laboratory results of the mother

Measure	d2	d9	d10	d16	d19	d20	d23	d25	d26
White blood cell count (3.5–9.5 × 10^9^/L)	2.7	NT	4.0	2.9	NT	3.9	NT	NT	5.3
Lymphocyte count (1.1–3.2 × 10^9^/L)	0.9	NT	1.2	0.8	NT	1.1	NT	NT	1.0
C-reactive protein concentration (0-6 mg/L)	0.1	NT	NT	1.0	NT	1.9	NT	NT	NT
**SARS-CoV-2 test by PCR**									
Nasopharyngeal swab	+	NT	+	NT	–	+	–	–	–
Feces	NT	NT	NT	NT	NT	–	NT	NT	NT
Serum	–	NT	NT	–	NT	–	NT	NT	–
Breast milk	–	–	NT	–	–	NT	NT	NT	NT
**SARS-CoV-2 antibody**									
Serum IgG	NT	NT	NT	+	NT	+	NT	NT	NT
Serum IgM	NT	NT	NT	–	NT	–	NT	NT	NT
Breast milk IgG	NT	+	NT	NT	NT	NT	NT	+	NT
Breast milk IgM	NT	–	NT	NT	NT	NT	NT	–	NT

*d2* hospital day 2, *NT* not tested

“+”, Positive

“-”, Negative

**Fig. 2:**
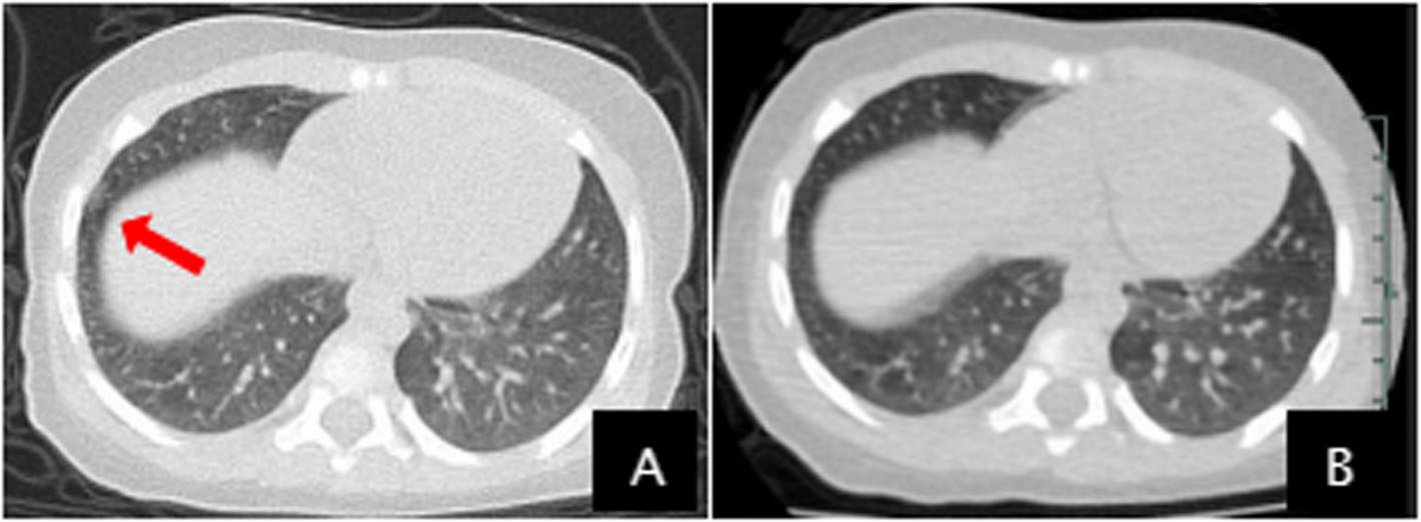
**a-b**: Chest CT plain scans of the child. **a**. Hospital day 4: ground-glass shadows in the lower lobe of the right lung; **b**. Hospital day 28: the ground-glass shadows in the lungs have basically absorbed

The patient’s 13-month-old son had a fever, occasional dry cough, and nasal congestion at admission to the hospital. The physical examination revealed a body temperature of 37.6 °C, respiratory rate of 23 breaths per minute, a pulse of 105 beats per minute, blood pressure of 95/56 mmHg, oxygen saturation of 99% while breathing ambient air, and bodyweight of 10 kg. There was no abnormality in lung auscultation. During the hospitalization, antiviral treatment with atomized inhalation of recombinant human interferon α-2b 1.5 million International Unit (IU) in 2 mL sterilized water was performed twice a day for 8 days, from day 1 to day 8 of hospitalization. On the day of admission, the white blood cell count was low at 3.7 × 10^9^/L, and lymphocyte count was 2 × 10^9^/L. The infant’s serum tested negative for SARS-CoV-2 IgG and IgM. On hospital day 2, the temperature returned to the normal value, and remained normal thereafter, but occasional dry cough and runny nose continued. On hospital day 4, feces and nasopharyngeal swab specimens were positive for SARS-CoV-2 nucleic acid, and plain chest CT scan revealed ground-glass shadows in the lower lobe of the right lung (Fig. 2a). On hospital day 6, the child’s cough was essentially resolved, while occasional rhinorrhea persisted. At that time, the counts of white blood cells and lymphocytes were 6.9 × 10^9^/L and 5.1 × 10^9^/L, respectively. The child became free of symptoms on hospital day 7. On hospital day 14, the serum was negative for SARS-CoV-2 nucleic acid, but positive for SARS-CoV-2 IgG and IgM. Nasopharyngeal swabs and feces specimens repeatedly tested positive for SARS-CoV-2 nucleic acid. On hospital day 28, when the child tested negatively two consecutive times for SARS-CoV-2 nucleic acid in nasopharyngeal swabs and chest CT indicated that the ground-glass shadows in the lungs were essentially absorbed (Fig. 2b), the child was discharged. The major laboratory results of the child are listed in Table 2.

**Table 2:** Clinical laboratory results of the child

Measure	d1	d4	d6	d14	d24	d26	d28
White blood cell count (4.0–12.0 × 10^9^/L)	3.7	NT	6.9	9.6	NT	NT	NT
Lymphocyte count (0.7–4.9 × 10^9^/L)	2.0	NT	5.1	6.4	NT	NT	NT
D-reactive protein concentration (0-6 mg/L)	1.5	NT	NT	0.1	NT	NT	NT
**SARS-CoV-2 test by PCR**							
Nasopharyngeal swab	NT	+	+	+	+	–	–
Feces	NT	+	NT	+	+	–	NT
Serum	NT	NT	NT	–	NT	NT	NT
**SARS-CoV-2 antibody**							
Serum IgG	–	NT	NT	+	NT	NT	NT
Serum IgM	–	NT	NT	+	NT	NT	NT

*d1* the day of admission, *NT* not tested

“+”, Positive

“-”, Negative

## Discussion

SARS-CoV-2 spreads mostly through droplets and close contact; both patients and asymptomatic carriers can be potential sources of infection [6, 7]. In the case presented here, the mother and her son came in close contact with a relative who developed fever 2 weeks later and was confirmed COVID-19. This family meeting caused an infection of at least eight persons. Based on the current data, the incubation period of COVID-19 pneumonia is 1–21 days, with an average of 5.2 days [8]. The patients mainly present with clinical symptoms such as fever, seldom dry cough, fatigue, nasal congestion, runny nose, sore throat, and diarrhea. Most patients had a good prognosis, and children had relatively mild symptoms. Among 425 early patients reported in the literature, there were no cases of children less than 15 years of age [8]. One reason for this finding could be associated with the presence of milder symptoms in children resulting in no testing or not listing them among confirmed cases. Patients occasionally present with a decreased or normal count of white blood cells, particularly lymphocytes. Typically, a chest CT scan shows multiple patchy opacities under the pleura, which subsequently progress to ground-glass opacities. In the current report, the incubation period was 12 days, and clinical manifestations, laboratory results, and imaging findings were consistent with general clinical features of COVID-19. Previous research found that the time from the onset of symptoms to recovery ranges from 12 to 32 days, but the test for SARS-CoV-2 nucleic acid on throat swabs was positive five to 13 days after discharge [9]. In the case reported here, the time from the onset of illness to discharge was as long as 28 days. During the hospitalization, the result of the test for SARS-CoV-2 nucleic acid changed from negative to positive, indicating that the patient isolation has to be continued after discharge to reduce the spread of the disease.

Some viruses in the mother can infect her offspring through breast milk [10]. Similar to SARS-CoV-2, Hepatitis C and Ebola viruses belong to the RNA viruses. It has been documented that small viral load of Hepatitis C and Ebola viruses can be detected in breast milk, raising the possibility that breastfeeding may result in mother-to-child transmission of the virus [11, 12]. At present there is no evidence demonstrating that SARS-CoV-2 can enter breast milk [3, 13]. Recently published data on nine breastfeeding women indicate that there was no SARS-CoV-2 in the colostrum [3]. In the present case, the tests for the presence of SARS-CoV-2 nucleic acid in the mother’s serum and milk were repeatedly negative, further confirming that the possibility of mother-to-child transmission via breast milk alone was very small, and breast milk is safe for direct feeding of infants.

Breast milk provides not only a variety of nutrients for infant growth and development but also many bioactive components, including antibodies, to provide protection against pathogenic microorganisms early in life [14]. The presence of IgM antibodies indicates the recent incidence of infection. IgG antibodies are an indication of possible immunity or resistance. SARS-CoV-2 exhibits species similarity to SARS-CoV-1. Research on antibodies against the SARS virus [15] demonstrated that SARS-CoV-1 IgM and IgG can be detected in the serum from the second week onwards. At week three, IgG can be detected with a 100% sensitivity and remains at a high level. IgM peaks at the acute stage of the disease, at approximately 3 weeks, and then disappears at week 12. IgG reaches its highest concentration at week 12, and maintains a high level for a long time, providing protection of patients from the recurrence of SARS. It appears the plasma of convalescent SARS patients may be effective in treating severe acute SARS patients, and suggests that SARS-CoV-1 IgG and/or IgM antibodies may represent passive immune antibodies capable of providing a protective or ameliorating effect against the invasion of the SARS-CoV-1. A follow-up study in China found that SARS IgG continues to be present for at least 3 years [16]. Circulating antibodies can enter the mother’s milk and be delivered to the offspring, providing them a passive immunity. A case was previously reported [17] of a pregnant woman that was infected with SARS at 19 weeks of gestation but subsequently recovered. SARS-CoV-1 antibody was present in her blood samples taken on days 12 and 29 after disease onset. She delivered a healthy baby at 38 weeks, and the SARS-CoV-1 nucleic acid was not detected in maternal and neonatal serum, nasopharyngeal swabs, placenta, umbilical cord blood, and amniotic fluid. However, the antibody against SARS-CoV-1 was detected in maternal serum, umbilical cord blood, and breast milk. In the case presented here, the patient’s milk was positive for SARS-CoV-2 IgG and negative for IgM on days 11 and 27 after the onset. On hospital day 14, the child’s serum was positive for both SARS-CoV-2 IgG and IgM, suggesting either breastmilk transfer to the infant or infant de novo production of the IgG and/or IgM, or both mechanisms. A protective antibody can be passed by breastfeeding from the mother to the offspring, preventing or decreasing the severity the children’s diseases.

Although the IgA in breastmilk is important for infants, the IgG shows higher concentration than the IgA and IgG is longer lived, and thus is preferred for serosurveillance studies [18]. Importantly, ELISA kits for IgA was not available at that time.

According to existing literature reports [3, 13, 19–21] vertical transmission from mother to child remains to be confirmed. In China, all infants were isolated from their mother with COVID-19 immediately after birth, and the baby was taking full formula feeds without breastfeeding. However, the WHO recommend that mother and infant should be enabled to remain together while rooming-in throughout the day and night and to practice skin-to-skin contact, including kangaroo mother care, especially immediately after birth and during establishment of breastfeeding, whether they or their infants have suspected or confirmed COVID-19 virus infection, and breastfeeding should continue up to 2 years of age or beyond [22]. Although most studies suggested the newborns had negative result for SARS-CoV-2 nucleic acid on nasopharyngeal swab specimens, one case reported pharyngeal swab for SARS-CoV-2 was positive at 36 h after birth when the mother wore a N95 mask during the Emergency Cesarean and had no close contact with the newborn [13]. During direct breastfeeding period, we cannot rule out the possibility of other contact transmission of newborn. Evidence from cases of COVID-19 positive mothers breastfeeding should be collected to confirm safety for uninfected children.

## Conclusions

In summary, this is one of the few reports of direct breastfeeding an infant by a mother with SARS-CoV-2 pneumonia. Observational studies suggest that breast milk is safe. Future studies should address the issue of detecting the level of SARS-CoV-2 IgG to assess the best time window for breastfeeding, and the risks vs benefits of direct breastfeeding.

## Data Availability

The dataset or transcripts are available from the corresponding author upon reasonable request.
